# Action Selection and Motor Decision Making: Insights from Transcranial Magnetic Stimulation

**DOI:** 10.3390/brainsci12050639

**Published:** 2022-05-12

**Authors:** Margherita Tecilla, Andrea Guerra, Lorenzo Rocchi, Sara Määttä, Matteo Bologna, Maria Herrojo Ruiz, Roberta Biundo, Angelo Antonini, Florinda Ferreri

**Affiliations:** 1 Department of Psychology, Goldsmiths, University of London, London SE146NW, UK; margherita.tecilla@gmail.com (M.T.); m.herrojo-ruiz@gold.ac.uk (M.H.R.); 2 IRCCS Neuromed, 86077 Pozzilli, Italy; andrea.guerra@uniroma1.it (A.G.); matteo.bologna@uniroma1.it (M.B.); 3 Department of Medical Sciences and Public Health, University of Cagliari, 09124 Cagliari, Italy; l.rocchi@ucl.ac.uk; 4 Department of Clinical and Movement Neurosciences, University College London Queen Square Institute of Neurology, London WC1N3BG, UK; 5 Department of Clinical Neurophysiology, Kuopio University Hospital, University of Eastern Finland, 70211 Kuopio, Finland; sara.maatta@kuh.fi; 6 Department of Human Neurosciences, Sapienza University of Rome, 00185 Rome, Italy; 7 Department of General Psychology and Study Center for Neurodegeneration (CESNE), University of Padua, 35131 Padua, Italy; roberta.biundo@unipd.it; 8 San Camillo IRCSS Hospital, 30126 Lido di Venezia, Italy; 9 Parkinson and Movement Disorders Unit, Study Center for Neurodegeneration (CESNE), Department of Neuroscience, University of Padua, 35131 Padua, Italy; angelo.antonini@unipd.it; 10 Unit of Neurology, Unit of Clinical Neurophysiology and Study Center for Neurodegeneration (CESNE), Department of Neuroscience, University of Padua, 35131 Padua, Italy

**Keywords:** action preparation, action selection, corticospinal excitability, motor decision making, motor cortex, movement, TMS

## Abstract

In everyday life, goal-oriented motor behaviour relies on the estimation of the rewards/costs associated with alternative actions and on the appropriate selection of movements. Motor decision making is defined as the process by which a motor plan is chosen among a set of competing actions based on the expected value. In the present literature review we discuss evidence from transcranial magnetic stimulation (TMS) studies of motor control. We focus primarily on studies of action selection for instructed movements and motor decision making. In the first section, we delve into the usefulness of various TMS paradigms to characterise the contribution of motor areas and distributed brain networks to cued action selection. Then, we address the influence of motivational information (e.g., reward and biomechanical cost) in guiding action choices based on TMS findings. Finally, we conclude that TMS represents a powerful tool for elucidating the neurophysiological mechanisms underlying action choices in humans.

## 1. Introduction

During daily life, goal-oriented motor behaviour relies on the estimation of the rewards/costs associated with alternative actions and on the appropriate selection of movements [1,2,3]. Motor decision making can be defined as the process by which a motor plan is chosen among a set of competing actions based on the expected value [4,5,6,7]. When multiple movement options are available, computing the predicted outcome for each action leads to competition among representations, with the most valuable response being implemented to optimise behaviour. For instance, when sitting at a table, we can either decide to reach for an apple or a peach according to our food tastes and/or the relative distance between the objects and ourselves [8]. Despite the relevance of decision making in influencing overt behaviour, action selection has been mainly studied in isolation for instructed/cued movements [9]. In such a scenario, the selection process is constrained by the perceptual features of the displayed cues, indicating the effector (i.e., any body part that responds to a stimulus; in the current context, a muscle of the upper/lower limbs) to activate for a successful performance in the absence of free choices. On these bases, in the following we will refer to action selection as the process by which a given effector is selected in response to specific perceptual stimuli (i.e., cued/instructed responses). On the other hand, we will refer to motor decision making to describe those situations in which choosing the action to execute depends on decision variables (e.g., estimated reward and cost associated with movements). The aim of this literature review is to provide an overview of the neurophysiological mechanisms of action selection and motor decision making in humans using evidence from transcranial magnetic stimulation (TMS).

Action selection and preparation have often been investigated in the context of choice reaction time (RT) tasks [9,10,11,12,13,14,15,16,17,18,19,20,21,22,23,24,25,26,27,28,29,30,31,32]. In such paradigms, participants are required to execute movements as fast as possible in response to imperative go signals. Choice RT paradigms evolve from simple RT tasks, where the same motor output (e.g., a left index finger press) is prompted in response to a unique go signal (e.g., a green circle). In choice RT tasks, two perceptually different imperative cues (e.g., right or left arrows) are displayed in separate trials to elicit the corresponding motor response (e.g., right or left index finger presses, respectively). The go cue is sometimes preceded by a warning signal (i.e., choice RT tasks with a delay), informing that the action has to be withheld for a certain amount of time [14,17,18,20,21,23,26,27,32]. Motor decision making has been studied by adapting choice RT tasks to include sensory uncertainty. Sensory uncertainty is introduced, for instance, by displaying both ambiguous and unambiguous imperative signals (e.g., red and blue signals prompting right and left finger movements, respectively (unambiguous trials); greyish cues possibly eliciting both right and left responses (ambiguous trials)) and manipulating the reward associated with the available motor responses (e.g., larger rewards for left finger movements) [33]. Along a similar line of thinking, asking subjects to choose between actions yielding different rewards (in terms of magnitude and probability) or having distinct biomechanical costs has also been pivotal for elucidating the mechanisms underlying motor decision making for free choices [34,35,36,37,38]. The principal aim of these paradigms consists in inferring the contribution of decision processes on motor behaviour by allowing participants to freely choose the motor response they expect to hold the largest value/smallest cost among a set of available options.

In the last two decades, TMS studies have contributed to gain a more comprehensive understanding of action selection and motor decision making by assessing changes in corticospinal excitability during choice RT tasks and value-based decision-making tasks. TMS is a non-invasive brain stimulation technique that involves delivering magnetic pulses through a coil positioned on the scalp [39,40]. Different TMS protocols, including single pulse (spTMS), paired-pulse (ppTMS), and dual-site TMS techniques allow to investigate corticospinal and intracortical excitability changes, as well as inter-hemispheric interactions. spTMS consists of delivering single TMS pulses on the scalp over the primary motor cortex (M1) and recording the resulting motor evoked potential (MEP) in the contralateral target muscle [40,41]. The MEP amplitude reflects the TMS-induced direct depolarisation of corticospinal cells originating in M1 and synapsing on spinal motor neurons. The electromagnetic pulse also activates the descending tract indirectly, via interfering with the intracortical, transcortical, and subcortical inputs. Therefore, the MEP amplitude not only results from the direct depolarisation of the corticospinal neurons, but also from the activity of additional circuits that indirectly input into the descending pathway, as well as from the spinal contributions [40,41]. When TMS is administered during voluntary muscle contraction, the MEP is typically followed by a temporary suppression of electromyographic activity, the so-called cortical silent period (CSP), which reflects motor cortex inhibition mediated by the GABA-ergic circuits’ activation [42]. Further insights into the activity of specific excitatory and inhibitory intracortical motor circuits can be gained by delivering two TMS pulses separated by a specific time interval (i.e., interstimulus interval, ISI) through the same coil over M1 (i.e., ppTMS) [43]. For instance, short-interval intracortical inhibition (SICI) is known to reflect the activity of the GABA_A_ interneurons within M1 and is assessed by delivering a subthreshold (conditioning) stimulus followed by a suprathreshold (test) stimulation at 1–6 ms ISI [43]. By observing the differences between conditioned (elicited by ppTMS) and unconditioned (elicited by spTMS) MEP amplitudes, it is possible to infer the effects of local cortical interneurons on the corticospinal tract.

Dual-site TMS implies delivering two pulses through separate TMS coils positioned over distant cortical regions and allows one to investigate the functional connectivity between areas, including the two in M1 (interhemispheric inhibition—IHI), the dorsal premotor cortex and M1 (PMd–M1), and the cerebellum and M1 (cerebellar-brain inhibition—CBI) [9,44]. IHI is mediated by transcallosal fibres acting on inhibitory GABA_B_ receptors and emerges when stimulating M1, before perturbing the contralateral M1 with a second suprathreshold TMS pulse (ISI of 10–50 ms), resulting in MEP suppression [45,46]. The PMd–M1 interaction is tested by applying the first pulse on the PMd and recording the ipsilateral MEPs elicited by stimulating the contralateral M1 with a second stimulus after 8–10 ms [47]. This transcallosal circuit entails significant hemispheric differences, leading to both MEP facilitation and inhibition [22]. CBI reflects the suppression of corticospinal excitability through the activation of inhibitory Purkinje cells and dentate-thalamus-cortical projections [48], and is assessed by applying a cerebellar hemisphere stimulation followed by TMS over the contralateral M1 after ~5 ms [49]. Overall, in dual-site TMS paradigms, the MEP amplitude elicited by the second test pulse (relative to an unconditioned MEP elicited by spTMS) informs on the contribution of pre-activated neurons in the target regions on corticospinal excitability.

All the above-mentioned TMS techniques have been extensively used to investigate the mechanisms underlying motor behaviour in humans [9,10,29,44]. TMS-based research has mainly elucidated the mechanisms supporting response selection and preparation of cued movements in choice RT tasks [11,12,13,14,15,17,18,19,20,21,22,23,24,25,26,27,28,30,31,32]. Yet, in the last decade, a number of TMS studies have given insight into the contribution of decision variables in shaping purposeful motor behaviour [33,34,35,36,38]. Thus, interpreting TMS findings on action selection for instructed movements and motor decision making allows to draw valuable inferences regarding the mechanisms regulating human action choices.

In the present literature review we will discuss TMS-based evidence on motor control, focusing on studies of action selection for instructed movements and motor decision making. In the first section, we will delve into the usefulness of the various spTMS, ppTMS, and dual-site TMS measures in characterising the contribution of motor areas and distributed brain networks to cued action selection. Then, we will address the influence of motivational information (e.g., reward and biomechanical cost) in guiding action choices based on TMS findings.

## 2. TMS in Action Selection

### 2.1. SpTMS

In choice RT paradigms, single TMS pulses (Table 1) are delivered at different timings after the onset of imperative signals and/or in the delay interval between go signals and preceding warning stimuli [9,10,12,13,14,15,16,17,18,19,21,23,24,26,27,28,29,31,32]. Muscles from which MEPs are recorded can be classified as selected, non-selected, or task-irrelevant, depending on their involvement in the forthcoming movement [16]. Selected muscles are activated to execute the wanted action (e.g., in the context of choice RT tasks for unimanual finger movements, trials with left-pointing cues, the left finger; in trials with right-pointing cues, the right finger); non-selected muscles are not required for executing the specific cued response, but may be called into action under certain conditions (e.g., trials with left-pointing cues, the right finger; trials with right-pointing cues, the left finger) and task-irrelevant muscles are unrelated to the action repertoire (e.g., in trials with pointing cues, the foot).

One of the most established findings is the progressive rise of the MEP amplitude elicited in the selected muscle, starting ~100 ms before movement onset [10,16,24]. Yet, recent work suggests that the observed facilitation is more likely to occur later, at around 30–60 ms prior to action [50]. Conversely, MEPs in non-selected effectors exhibit a gradual suppression during movement preparation, with MEPs for selected and non-selected muscles diverging 60–100 ms before movement onset [10,16,24,28]. Excitability differences between motor effectors are also quantifiable by looking at the duration of the CSP elicited by TMS. As the movement draws nearer, the CSP duration decreases in selected muscles and increases in non-selected effectors, with significant differences emerging ~80 ms after the onset of the imperative signal [12,31,51]. Thus, movement selection is shaped by the increased excitability in selected muscles and the suppression of non-selected effectors. Preparatory inhibition has been considered within the competition resolution framework, according to which unwanted motor alternatives are gradually eliminated through neural competition to favour the execution of the appropriate response [14,16]. Competition between action plans is context-dependent, whereby suppression is constrained by the degree of similarity between options (anatomical similarity/distance-dependent) and the degree to which alternative motor representations have been engaged in the past (experience-dependent) [23]. However, a comparable inhibitory trend is also detected in the MEP generated by non-selected task-irrelevant muscles [18]. This finding cannot be easily reconciled with the competition resolution hypothesis as, based on the proposed mechanism, inhibition should only occur in cortical motor representations of muscles anatomically or contextually linked to the selected effector. TMS-based research has supported the existence of an additional broad and non-specific inhibitory mechanism, by which all motor representations are inhibited, regardless of their involvement for the forthcoming movement. This framework has been referred to as the motor gain hypothesis, where global inhibition serves to increase the signal-to-noise ratio, favouring motor selection and preparation [16,18,52].

Finally, it is worth noting that the excitability of the corticospinal output to selected effectors is transiently inhibited (i.e., in a choice RT task with a delay, during the interval between the warning cue and go stimulus; in the absence of a delay period, at the earliest stages of movement preparation), before increasing close to movement onset. Interestingly, this initial suppression is stronger in selected compared to non-selected muscles [14,15,17,18,21]; however, see 27 for the inconsistent inhibition of the selected effectors across the variants of the instructed-delay choice RT task]. The function of this suppression has been first interpreted in the context of the impulse control framework, which postulates a role of preparatory inhibition in preventing premature movements eruption when actions need to be withheld. According to this view, the greater suppression in selected effectors, compared to non-selected muscles, reflects a higher degree of inhibition to prevent early action release. Notably, the observed peripheral suppression does not seem to be reflected in decreased cortical activity in the corresponding M1 region. Thus, programs for motor actions are activated at the cortical level to support action preparation, while the peripheral engagement is inhibited through spinal suppression to prevent impulsive movement generation [16,17]. However, it should be noted that other findings challenge this perspective. Hannah and colleagues [19] suggested that preparatory inhibition in cue-guided movements only pertains to a specific set of excitatory inputs to corticospinal neurons, instead of being global, and that greater suppression leads to faster RT. The latter outcome implies that selective inhibition of a specific set of motor cortical neurons might be a fundamental aspect for successful movement preparation, rather than being involved in preventing the release of unwanted movements. Further support to this notion comes from the study by Ibáñez and co-workers [50], where preparatory inhibition was detected in selected muscles even during a spontaneous motor response. This finding is at odds with the impulse control theory as, according to the latter, the inhibition of effectors should only be expected when there is the possibility of prematurely releasing a motor plan. Rather, the presence of suppression before spontaneous and uncued movements, supports the notion of preparatory inhibition as a general feature of primary motor cortex physiology [50].

In spTMS studies on RT tasks, right-handed participants show greater suppression for the right non-selected muscles after left-M1 stimulation, compared to the left non-selected muscles after right-M1 stimulation [24]. These results have been subsequently expanded by Klein and colleagues [21], who clarified that the stronger inhibition in the right non-selected muscles was preceded by an initial increase in excitability. Conversely, MEPs elicited in the left non-responding muscles displayed a milder and more constant suppression. The initial enhanced excitation in the right non-selected effectors could reflect the involvement of motor areas in both the right and left hemisphere when planning actions with the left hand, while the later-occurring greater suppression might result from higher inhibitory demands when inhibiting unwanted right-side movements [21]. Hemispheric differences also emerge during the delay period when analysing changes in corticospinal excitability for selected muscles [21,26,32]. Indeed, Poole and colleagues [26] found that when preparing non-dominant left finger movements, ipsilateral MEPs are facilitated. Surprisingly, for movements executed with the dominant right hand, ipsilateral MEPs are unchanged. MEPs in the non-selected hand are always inhibited. The absence of increased excitability in right selected muscles could indicate a greater baseline activation of the left hemisphere, which therefore does not require further excitation when planning an upcoming right-side action [26]. To execute movements with the left hand, this asymmetry must be reversed by suppressing the activation of the dominant left hemisphere (controlling for non-selected right effectors) and boosting the excitation of the right hemisphere (activating selected left-side muscles) ([26]; refer to the original paper for the discussion about the absence of the impulse control effect). Overall, these findings point towards a dominant role of the left hemisphere in action selection and preparation [21,24,26,28].

More recently, directional spTMS has been applied to delve into the different contributions of finer neural mechanisms underlying response selection and preparation [19,44]. During the preparatory period of choice RT tasks, MEPs induced by anterior–posterior TMS (TMS_AP_) are more strongly suppressed in both selected and non-selected muscles, compared to MEPs elicited by posterior–anterior TMS (TMS_PA_) [19]. As TMS_AP_ probes polysynaptic excitatory inputs to pyramidal tract neurons, this indicates that preparatory inhibition is at least partially supported by the suppression of cortical polysynaptic circuits. Importantly, because TMS_PA_ triggers excitatory monosynaptic inputs on corticospinal neurons, the reduced MEP_PA_ inhibition is consistent with the idea that the suppressed polysynaptic circuits (as detected with TMS_AP_) are counterbalanced by an increased activation of monosynaptic influences on cortical output neurons [19,44]. Thus, findings from directional spTMS seem to support a different functional contribution of specific neural populations in action preparation, whereby the selection process relies on the synergistic inhibition and excitation of polysynaptic and monosynaptic inputs, respectively [44].

To conclude, spTMS provides valuable insights into the role of neurophysiological mechanisms supporting action selection and the preparation of cued responses. Along with excitation in selected muscles, multiple non-mutually exclusive inhibitory mechanisms shape the selection process [16,50]. In addition, spTMS findings highlight hemispheric asymmetries in action selection, pointing towards a dominant role of the left hemisphere [21,24,26,28]. More recent, state-of-the-art spTMS protocols have expanded this knowledge by delving into the different contributions of distinct neural populations in action selection and preparation, providing important insights into the role of M1 in cued choices [19,44].

### 2.2. PpTMS

Despite the ppTMS potential to target a variety of intracortical circuits, the literature on intra-M1 facilitatory mechanisms on action selection is scarce. Moreover, methodological features of ppTMS (i.e., ISI timings) constrain its applicability to choice RT tasks, limiting the area of investigation to short-interval intracortical processes [9,44]. Thus, most ppTMS studies have focused on SICI, addressing the contribution of inhibitory GABA_A_ interneurons in cued action selection (Table 2).

One of the most established findings is SICI decrement in selected muscles, starting ~75 ms before movement onset [30,53]. Release from intracortical inhibition is, therefore, likely to assist the build-up of the corticospinal excitability required for action execution. Yet, no significant strengthening of SICI is detected in non-selected effectors as the movement draws nearer [30]. Moreover, a release of SICI in selected muscles is also observed in the time interval between the warning and go signals, suggesting that other suppressing mechanisms beyond SICI are likely to support the inhibition of non-selected muscles and impulse control mechanisms during action withholding [17]. The relevance of intra-M1 inhibitory mechanisms in action preparation is also supported by clinical research showing abnormal SICI modulation in patients with movement impairment (e.g., Tourette syndrome, stroke) [54,55]. It is worth noting that recent work has highlighted differences in SICI depending on conditioning pulse intensities [56]. In fact, low-intensity pulses might only perturb a restricted portion of GABA_A_ interneurons, providing a distorted picture of the inhibitory intracortical circuits supporting action control. However, conditioning at a higher intensity might recruit excitatory interneurons, whose effects overlap with the inhibition [57]. Future studies should therefore aim to deliver conditioning pulses at varying intensities to control for the potential confounding effect of the mixed recruitment of excitatory and inhibitory input cells to pyramidal tract neurons [56].

### 2.3. Dual-Site TMS

Dual-site TMS paradigms have further expanded our understanding of preparatory mechanisms in guiding action selection (Table 2). To date, three circuits have been mainly targeted: M1–M1, PMd–M1 and, to a lesser extent, cerebellum–M1 (see also [58] on the role of the dorsomedial parieto-motor circuit in grasping movements).

The inhibitory interplay between bilateral M1s can be evaluated by stimulating both cortices through separate coils at specific ISIs [45,46]. Empirical evidence suggests that decreased IHI targets the hemisphere contralateral to the selected effector, starting ~90 ms before movement onset. Conversely, IHI towards M1, ipsilateral to the selected effector, is maintained with constant during movement preparation [20,59,60]. Interestingly, IHI is affected by the amount of information provided by the warning cue preceding the go signal [20]. For cues which are not informative about the forthcoming movement, a global release from inhibition is detected at the imperative signal onset and later during the reaction period in both selected and non-selected effectors, which is likely to reflect a non-specific mechanism for action readiness. On the other hand, when warning signals reliably inform about the upcoming action to execute, only the selected hand is released from IHI [20]. Thus, as for SICI, the release of IHI indicates that this mechanism is likely to act independently from the inhibitory changes occurring in corticospinal excitability during action selection and preparation observed with spTMS. Importantly, dual-coil paradigms probing IHI have highlighted hemispheric asymmetries in action initiation, with right-hand movements requiring a greater disinhibition of the contralateral M1 compared to left-hand actions [61]. According to the authors, the higher flexibility in IHI modulation observed for right hand movements is likely to assist in a more precise and fine-tuned performance of dominant side responses in right-handed subjects. Overall, IHI protocols emphasise the relevance of inhibitory M1–M1 interplay in shaping action selection for cued responses. The synergistic release and maintenance of inhibition in selected and non-selected muscles, respectively, is likely to reflect a fundamental requirement to perform accurate unimanual movements and avoid unwanted mirror activity in resting effectors.

PMd–M1 protocols assess the interaction between these areas through transcallosal pathways, thus allowing to investigate the functional contribution of PMd in shaping the selection of cued unimanual movements. A facilitatory effect of PMd on MEPs for externally cued actions is detected ~75 ms after the go signal [11,22,25]. In addition, transcallosal inhibition ~100 ms after imperative signal onset is registered over non-selected muscles which are ipsilateral to the stimulated PMd [22]. Notably, Koch and colleagues highlighted important hemispheric differences in the PMd–M1 interplay: despite both PMd-inhibiting ipsilateral non-selected muscles, only the left PMd leads to MEPs facilitation of the ipsilateral selected effectors. In fact, conditioning the left M1 through right-PMd perturbation does not result in MEPs facilitation in the selected right effector, despite leading to MEPs suppression when a left-hand movement is prompted. Action selection is therefore likely to be assisted by asymmetrical transcallosal interactions between the PMd and M1, with the left PMd facilitating ipsilateral movements and the bilateral PMd suppressing non-selected muscles. Importantly, the inhibitory effect of the PMd over M1 has also been detected in the delay period of choice RT tasks, with left PMd stimulation leading to the suppression of ipsilateral-selected effectors to prevent premature movement eruption [62]. Overall, these findings show that changes in the PMd–M1 interplay are likely to reflect early processes to guide action selection, whereby cued movements are favoured at the expense of alternative motor programs. Furthermore, the described hemispheric asymmetries are consistent with TMS evidence supporting a dominant role of the left hemisphere in fine-tuning action selection and preparation [21,24,26,28].

CBI studies have allowed to test whether action planning is influenced by activity in cerebellum–thalamus–M1 projections. CBI is inhibitory at rest, as indicated by reduced MEP amplitudes when conditioning M1 with a contralateral pulse over the cerebellum. When preparing to move, CBI is gradually reduced, with the greatest release from inhibition being detected at movement onset [9,44]. Of note, Kassavetis and colleagues [63] failed to find a muscle-specific CBI effect, as the inhibitory reduction not only was observed in selected muscles, but also in surrounding effectors. These results were challenged by Spampinato and co-authors [64], who found CBI decrement limited to selected muscles, but not in task-irrelevant ones. Yet, it has to be noted that the above-mentioned studies differ significantly in terms of methodology (e.g., irrelevant muscles are anatomically close to and far from selected ones in [63] and [64], respectively), which could account for the observed contrasting results. Thus, whether CBI is specifically involved in action selection through the disinhibition of selected effectors, or more broadly in action initiation, is still poorly understood. Future research should look into the specific role of CBI in cued action selection by coupling dual-coil TMS protocols with choice RT tasks [44].

## 3. TMS in Value-Based Motor Decision Making

One of the main factors influencing motor choices is the potential gain/cost associated with actions [8]. By perturbing M1 activity through TMS it is possible to understand how decision processes influence the motor system to guide goal-oriented behaviour [10].

Evidence from spTMS studies suggests that appetitive stimuli increase cortical excitability in selected effectors whereas aversive cues lead to the opposite outcome, decreasing MEP amplitude [65,66]. More recent work assessed the temporal dynamics of these modulatory influences on corticospinal excitability [67]. The authors used a choice RT paradigm with a delay coupled with TMS over the left M1, whereby single pulses were delivered at different timings in the interval between the cue and target onset. The cue informed about whether a reward (binary: 1 point; 0 points) could be obtained upon a successful performance, while the target colour indicated the type of movement required (i.e., left vs. right finger movements). Compared to no-reward trials, the MEPs amplitude in incentivised trials increased at the first stimulation time point (400 ms after cue onset), to then decrease progressively, with maximal suppression peaking just before approaching the appropriate time for movement execution (800 ms after cue presentation). The observed early reward-driven increase in excitability is in line with previous research indicating greater tendencies towards action in response to motivational cues [68]. The subsequent progressive decrease might be instead contextualised within the impulse control framework, according to which inhibition of potentially selected effectors prevents premature movement onset [16,17].

Additional TMS studies (Table 3) have specifically addressed the interplay between decision making (e.g., expected reward and cost associated with alternative movements) and action-based processes, clarifying the mechanisms supporting motor decision making for free choices [33,34,35,36,38].

Within this framework, Klein and colleagues [33] delivered single TMS pulses over the right M1 at different time points (i.e., baseline, imperative signal, movement preparation) in a choice RT task under sensory uncertainty, where participants were instructed to either move the left or right index finger according to the colour of imperative signals to receive a reward. Of note, the authors manipulated the saturation of the go signals, whereby subjects were presented with both unambiguous (i.e., the colour of the stimulus was clearly distinct—red stimuli triggered a right finger response, blue stimuli instructed a left finger response) and ambiguous stimuli (i.e., the colour of the go signal was less distinguishable—greyish stimuli could either trigger a right or left finger response) in different trials. They also varied the amount of reward given for left and right index movements (i.e., in reward_neutral_, the compensation was equal for both hands; in reward_biased_, the compensation was greater for left-hand responses). Results showed that more left finger movements were performed in the reward_biased_ condition, compared to reward_neutral._ Strikingly, left MEPs measured at the imperative signal onset were larger in the reward_biased_ condition, compared to reward_neutral_, suggesting that greater reward expectations upregulated the initial motor activity. Additionally, the reward-driven effect on the MEP amplitude during movement preparation was influenced by how informative the imperative signal was, with corticospinal facilitation increasing more for ambiguous signals, compared with clearly distinguishable ones. This has been explained by considering that, in ambiguous trials, the decision about which finger to move was highly driven by reward expectation, as opposed to obvious trials, wherein action choice depended entirely on clearly distinguishable stimuli colours. Thus, rewards synergistically shifted the starting point and the mean rate of the accumulation of motor activity for forthcoming actions. Notably, the greater the reward-induced effect on corticospinal excitability 100 ms after ambiguous imperative signals’ onset, the larger the proportion of left-side responses. Thus, this study has demonstrated that motivational information acts on the motor system to shape the response choice, expanding knowledge on the relationship between decision- and action-based processes.

Klein-Flügge and Bestmann [36] made a step forward by delving into the temporal dynamics of the relationship between decision making and motor choices. The authors coupled spTMS to computational modeling in the context of a value-decision task in which participants had to decide to either execute a right or left finger button press (i.e., choice trials). Each alternative was associated with a different reward probability and magnitude, with participants aiming to gain the highest winnings amount. As a control condition, in some trials, subjects were instructed to use a specified effector to receive the incentive in the absence of choice (i.e., forced choice trials). Single TMS pulses were applied on the left M1 at six different timings in the interval between stimuli presentation and movement onset. Behavioural data were then modeled with the cumulative prospect theory (see [36] for further details) to compute the expected subjective value for each of the two options in choice trials. As expected, data revealed larger right MEPs for right-side movements, compared to left-hand responses. Strikingly, response-locked analyses showed that differences in corticospinal excitability for selected (i.e., right movements) and non-selected (i.e., left movements) muscles emerged earlier in choice compared to forced-choice trials (510 ms and 330 ms before movement eruption, respectively). To corroborate this finding, the authors performed more conservative analyses by isolating the decision period, calculated as the interval between trial onset and the time of the first corticospinal excitability divergence for right and left movements in forced-choice trials (i.e., 38% of RT). Still, MEPs differences between the selected and non-selected effectors emerged during the decision period in the choice trials. These data suggest that the motor bias supporting action competition in the choice trials occurred before the termination of the decision process, supporting a temporally parallel interplay between action-based processes and decision making. Moreover, corticospinal excitability in the choice trials varied as a function of the expected value difference for alternative responses, suggesting that changes in motor excitability can provide insights into how valuable (in terms of reward) an action is, compared to its response alternative.

Having clarified the role of motivational information on M1 activity, Mooshagian and colleagues [38] disentangled the different contributions of reward probability (p_reward_) and outcome uncertainty on corticospinal excitability. Uncertainty about the outcome varies as a function of reward probability, being minimal for p_reward_ = 1 and maximal for p_reward_ = 0.5. In their experiment, participants were told to either find or avoid a target (i.e., a circle) hidden behind a shaded square to earn a fixed trial-wise monetary reward. At each trial, a single shaded square, an empty square, or two shaded squares were displayed. Varying the number of stimuli presented (i.e., one vs. two squares) and their visual features (i.e., empty vs. shaded squares) the authors manipulated the reward probability (i.e., 1, 0.5 or 0) and outcome uncertainty (i.e., 0 or 0.5). As subjects were instructed to alternately find or avoid the hidden target, differences in task framing were also considered. spTMS was delivered 250 ms following stimuli presentation on the left M1 and, after an additional 750 ms, participants were required to express their response (i.e., either left index or middle finger movement when two squares were displayed; middle finger in response to single squares). Results showed that MEPs linearly increased with reward probability when subjects were asked to find the hidden target, with a greater reward probability corresponding to larger MEPs. Yet, no MEPs changes were found across the reward probabilities in the avoid condition. Importantly, varying the degree of outcome uncertainty did not result in MEPs modulation. Additionally, the authors evaluated the potential contribution of reward probability on SICI through ppTMS, yet no significant effects emerged. These findings showed that corticospinal excitability is sensitive to the task framing (find vs. avoid) and is likely to reflect changes in reward probability, rather than outcome uncertainty.

Overall, these results challenge traditional serial approaches to response choice, according to which motor involvement only occurs after a decision has been made [69]. TMS findings are indeed consistent with more recent action-based models suggesting that decision processes solely terminate once the movement is complete, with action representations in the motor cortex and decision making influencing each other throughout movement selection and preparation [36,38,69,70]. Similar conclusions have also been drawn in the context of spTMS coupled with a perceptual decision task, whereby participants were instructed to execute distinct unimanual movements in response to the gender of human face pictures (i.e., pinch or grip actions for female or male faces, respectively) [71]. Muscle activity for pinch and grip movements was recorded at different time points after stimulus onset and task difficulty was manipulated by varying the ambiguity of the faces’ gender. Data showed that MEP amplitudes mirrored the accumulation of decision information with greater perceptual uncertainty, leading to more temporally sustained motor perturbation (i.e., earlier divergence in MEPs elicited between selected and non-selected effectors for ambiguous vs. clearly distinguishable faces). Although this study did not address the influence of motivational information on action choice, therefore limiting its relevance for the current literature review, it reinforces the crucial role of motor activity in decision processes.

Besides rewards, additional motivational variables exert a relevant role in action choice, such as the estimated effort associated with response options. Cos and colleagues [34] implemented a decision task where participants were asked to execute reaching movements towards one of two displayed targets. In each trial, the effort for computing alternative responses varied in terms of both their biomechanical cost (i.e., degree of muscle torques and hand displacement) and path distance (i.e., length of reaching movement from the origin to the target). Single TMS pulses were delivered over M1 at different timings after stimulus onset and MEPs were recorded from six contralateral muscles. Consistent with previous behavioural findings [72], the results showed that subjects tended to prefer the least effortful action. Importantly, the predicted cost associated with action alternatives was reflected in corticospinal excitability changes, with MEPs amplitude diverging as early as 150 ms after stimulus onset for competitive movements (i.e., larger MEPs for a less effortful movement; this relationship is inverted later in the trial to reflect biomechanical requests). This suggests that action cost is computed very quickly in the brain, biasing motor activity early in the decision process. Thus, not only the reward, but also the cost associated with motor options drives competition between action representations in the motor system to influence decision making.

Finally, in a recent paper, the involvement of the motor cortex for computing hasty (i.e., high speed, low accuracy) and cautious (i.e., low speed, high accuracy) motor decisions has been evaluated [35]. The authors applied spTMS bilaterally over M1 to elicit finger and leg MEPs while participants chose between right and left index movements. Deciding which finger to move required participants to predict which circle, among two lateral options, was about to receive the highest number of tokens. Importantly, incorrect responses led to either a high or low penalty, prompting cautious or hasty decisions, respectively. On the other hand, correct choices led to a reward, which decreased throughout the experiment, therefore favouring hasty decisions. Results showed that hasty motor choices were supported by a broad MEPs facilitation in the selected (i.e., index) and remote irrelevant (i.e., leg) muscles on the chosen side. Yet, this effect was accompanied by a local MEPs suppression in irrelevant muscles that were anatomically close to the selected index finger (i.e., thumb and pinky muscles). No modulatory effects were detected on the unchosen side. Hence, when the urge to move is high, the decision process is supported by two superimposed modulatory mechanisms involving the responding body side, namely a broad facilitation together with a local suppression of those representations close, in terms of somatotopy, to the selected muscle. These novel findings reinforce the relevance of M1 when it comes to generating motor decisions, as well as the role of context-dependent properties (e.g., time pressure) in affecting corticospinal excitability.

Overall, evidence consistently shows that in a free choice scenario with multiple motor options available, people tend to execute the most rewarding/least effortful movement. By coupling behavioural paradigms with spTMS, it is possible to infer how motivational information acts on the motor system to bias motor decisions, with MEPs amplitude reflecting changes in the predicted value for forthcoming alternative actions. Moreover, spTMS has also clarified the temporal dynamics regulating the interplay between decision making and action control, revealing that competition among movement representations in the motor system already takes place during the decision period. This supports the notion of temporally parallel decision and motor processes, highlighting a crucial role of the motor cortex in action choice.

## 4. Conclusions and Future Perspectives

In the present literature review we have discussed a variety of TMS protocols to gain a more comprehensive understanding of the mechanisms supporting the selection of instructed responses and motor decision making.

In daily life, motor execution relies on computing the expected value associated with different motor options and selecting the appropriate responses [1,2,3]. Despite the fundamental contribution of decision variables in shaping goal-oriented motor behaviour, TMS protocols on action selection have mainly investigated the neurophysiology underlying cued responses in the absence of free choices. Consistent with this, spTMS has shown that the selection and preparation of instructed actions relies on facilitatory and inhibitory corticospinal mechanisms directed to selected, non-selected, and task-irrelevant effectors, providing important information about the functional significance of preparatory inhibition [16,50].

Further, spTMS protocols have highlighted a dominant role of the left hemisphere in movement selection and preparation, which likely explains the greater accuracy of right-hand movements in right-handed individuals [21,24,26,28]. More recently, spTMS protocols have also explained the different contributions of inhibitory polysynaptic and excitatory monosynaptic inputs to corticospinal tract neurons for fine-tuning response selection and preparation [19,44]. ppTMS and dual-site TMS allowed the characterization of additional neurophysiological modulatory processes underlying action selection, including SICI, IHI, PMd–M1, and CBI [9,11,20,22,25,30,56,59,60,61,62,63,64]. Overall, activity in local M1 circuits and diffuse brain networks affects the selection process through overlapping facilitatory and inhibitory mechanisms. Specifically, the modulation of transcallosal connections between bilateral M1s and PMd–M1 appears to be a key mechanism for the selection of instructed unimanual movements, minimising the risk of contralateral mirror activity in resting effectors. It is, nonetheless, worth noting that ppTMS applicability in the context of choice RT tasks is significantly constrained by the experimental design, limiting inferences on how long intracortical inhibitory and facilitatory mechanisms could support the appropriate selection of motor responses [9,44].

When not limiting action selection to cued discrete choices, TMS has also provided valuable insights into the interplay between decision variables (e.g., reward and cost of potential movements) and motor activity [10,33,34,35,36,38]. The prospect of obtaining rewards influences motor choices and biases corticospinal excitability. In fact, when executing an action over its alternative is more likely to lead to incentives, MEPs recorded on the corresponding selected muscle are upregulated compared to neutral conditions. Moreover, this effect correlates with behavioural preferences, demonstrating the contribution of reward-driven effects on cortical excitability in shaping action choices [33]. spTMS has also elucidated the temporal dynamics of motor decision making, supporting a model of parallel (and not sequential, as previously thought) development of decision- and action-based processes [34,36,38].

This literature review was intentionally focused on the neural temporal dynamics characterising action selection and motor decision making, therefore neglecting repetitive TMS (rTMS) approaches, which are more tailored to neuromodulation purposes. Furthermore, it is worth mentioning that inferences from TMS protocols are intrinsically constrained by MEPs not only being influenced by cortical mechanisms, but also by spinal excitability [73]. In order to overcome this methodological limitation and record direct measures of brain activation, TMS has been more recently combined with electroencephalography (EEG). TMS-EEG allows one to record EEG responses to TMS with a high temporal resolution, providing valuable insights into cortical excitability and cortico-cortical effective connectivity mechanisms [73,74,75,76,77]. Coupling the TMS-EEG methodology with motor tasks could, therefore, expand our knowledge about the neural mechanisms supporting action selection and motor decision making by looking at the trial-by-trial spatio-temporal distribution of TMS-evoked potentials in the EEG signal.

To conclude, TMS represents a powerful tool for elucidating the neurophysiological mechanisms underlying the selection of cued motor responses. Over the last decade, TMS has found valuable applications beyond response selection in isolation, and has given insights into the synergistic interplay between decision- and action-based processes, thus providing a more comprehensive understanding of action choices in humans.

## Figures and Tables

**Table 1:** Characteristics of key studies using spTMS in the context of choice RT tasks.

Reference	Delay Period (Duration—Informativeness of the Warning Cue)	Task-Related TMS Timings	TMS Location	Main Findings and Elements of Novelty
Leocani et al. (2000) [24]	No	Between 20 and 400 ms after go signal	vertex	RP: MEPs facilitation in selected muscles; MEPs suppression in non-selected muscles; left hemispheric dominance for movements
Burle et al. (2002) [12]	Yes (1000 ms—uninformative)	1/4, 1/2, 3/4, and the whole first decile of individual RT distribution	left M1	RP: increase CSP duration in non-selected muscles; decrease CSP duration in selected muscles
Duque & Ivry (2009) [17]	Yes (between 900 and 1200 ms—informative and uninformative)	800 ms after warning cue + 70 ms before individual RT	right M1	DP: stronger MEPs inhibition in (potentially) selected muscles than non-selected muscles
Duque et al. (2010) [14]	Yes (between 900 and 1200 ms—uninformative, partially and fully informative)	100, 800 ms after warning cue + 50, 100, 150, 200, 250 ms after go signal	right M1	DP: MEPs inhibition in (potentially) selected muscles and non-selected muscles, but not irrelevant muscles
Tandonnet et al. (2012) [31]	Yes (500 or 2500 ms—uninformative)	Go signal + 6 timings between 60 ms after go signal and the first decile of individual RT distribution	left M1	RP: increase CSP duration in non-selected muscles; decrease CSP duration in selected muscles
Duque et al. (2014) [15]	No and Yes (900 ms—uninformative)	890 ms after warning cue + 50, 100, 150, 200, 250 ms after go signal	right M1	RP: transient MEPs inhibition in selected muscles (inhibition in selected muscles not restricted to the delay period of choice RT tasks)
Labruna et al. (2014) [23]	Yes (900 ms—informative)	800 ms after warning cue	right M1	DP: MEPs inhibition in selected muscles; MEPs inhibition in non-selected muscles is constrained by anatomical and/or functional similarity
Greenhouse et al. (2015) [18]	No and Yes (900 ms—informative)	800 ms after warning cue + 150 ms after go signal	right M1	DP: MEPs inhibition in selected muscles, non-selected muscles and irrelevant muscles
Klein et al. (2016) [21]	No and Yes (500 ms—partially and fully informative)	450 ms after warning cue + 75, 125, 175, 225, 275 ms after go signal	right and left M1	DP: similar inhibitory changes in left and right M1 RP: constant and milder inhibition of MEPs in left non-selected muscles; initial facilitation and later stronger inhibition of MEPs in right non-selected muscles; left hemispheric dominance for movements
Quoilin et al. (2016) [27]	Yes (between 1000 and 1200 ms—informative)	950 ms after warning cue	right and left M1	DP: MEPs changes in selected muscles are sensitive to task design
Hannah et al. (2018) [19]	Yes (500 ms—uninformative)	Warning cue + 250 ms after warning cue + go signal + 35%, 70% of mean RT	left M1	DP and RP: MEPs inhibition pertains to a specific set of excitatory inputs, instead of being global; greater inhibition leads to faster RT
Poole et al. (2018) [26]	No and Yes (500 ms—informative)	200, 300, 400 ms after warning cue	right and left M1	DP: unchanged MEPs in dominant selected muscles, MEPs inhibition in non-dominant non-selected muscles; MEPs facilitation in non-dominant selected muscles, MEPs inhibition in dominant non-selected muscles; effects are sensitive to task experience
Quoilin et al. (2019) [28]	No	Go signal + 80, 130, 250, 300, 350 ms after go signal	right and left M1	RP: MEPs facilitation in selected muscles; unchanged MEPs in non-selected muscles; MEPs inhibition in irrelevant muscles of the non-responding hand

ISI = interstimulus interval; RT = reaction time; RP = reaction period; DP = delay period; M1 = primary motor cortex; CSP = cortical silent period; MEP = motor evoked potential; Informative warning cue = the cue informs about the required response for the forthcoming movement; Uninformative warning cue = the cue does not inform regarding the required response, the response is only indicated by the go signal. Note: Studies are listed in chronological order; The absence (No) and presence (Yes) of a delay period within the same publication refers to distinct experiments/trial type; When several experiments belong to the same publication, TMS timings are merged across experiments; Only task-specific TMS timings during the delay or reaction periods are reported—information for TMS delivered during baseline or rest is not included; For each publication, only key/novel findings are reported.

**Table 2:** Characteristics of key studies using ppTMS and dual-site TMS in the context of choice RT tasks.

Reference	Brain Mechanism Targeted	ISI (ms)	Delay Period (Duration—Informativeness of the Warning Cue)	Task-Related TMS Timings	TMS Location	Main Findings and Elements of Novelty
Koch et al. (2006) [22]	PMd–M1 *	8	Yes (between 1000 and 3000 ms—uninformative)	50, 75, 100, 125, 150, 200 ms after go signal	CP: left (right) PMd TP: right (left) M1	RP: left PMd facilitates MEPs in left selected muscles and suppresses MEPs in left non-selected muscles; right PMd suppresses MEPs in right non-selected muscles
Boorman et al. (2007) [11]	PMd–M1	8	No	50, 75, 100 ms after go signal	CP: left (right) PMd TP: right (left) M1	RP: PMd facilitates MEPs
O’Shea et al. (2007) [25]	PMd–M1*	8	No	50, 75, 100, 125, 150 ms after go signal	CP: left (right) PMd TP: right (left) M1	RP: PMd facilitates MEPs; absence of hemispheric asymmetries in PMd–M1 interactions
Duque & Ivry (2009) [17]	SICI	3	Yes (between 900 and 1200 ms—informative and uninformative)	800 ms after warning cue	right M1	DP: SICI release in selected muscles; unchanged SICI in non-selected muscles
Soto et al. (2010) [30]	SICI	2.5	Yes (between 500 and 1800 ms—uninformative)	Go signal + 125, 100, 75, 50, 25 ms before individual RT	left M1	RP: SICI release in selected muscles; unchanged SICI in non-selected muscles
Hinder et al. (2018) [20]	IHI	10, 40	Yes (500 ms—informative and uninformative)	Warning cue + go signal + 25%, 50%, 80% of individual RT	CP: left M1 TP: right M1	RP: IHI (ISI10) release in selected muscles and non-selected muscles for uninformative warning cues; IHI (ISI10) release in selected muscles and unchanged IHI in non-selected muscles for informative warning cues. Effects are sensitive to ISIs

ISI = interstimulus interval; RT = reaction time; RP = reaction period; DP = delay period; M1 = primary motor cortex; PMd = dorsal premotor cortex; MEP = motor evoked potential; SICI = short-interval intracortical inhibition; IHI = interhemispheric inhibition; CP = conditioning pulse; TP = test pulse; * = other ppTMS techniques are used in the study, in addition to the one reported; Informative warning cue = the cue informs about the required response for the forthcoming movement; Uninformative warning cue = the cue does not inform regarding the required response, the response is only indicated by the go signal. Note: Studies are listed in chronological order; When several experiments belong to the same publication, TMS timings are merged across experiments; Only task-specific TMS timings during the delay or reaction periods are reported—information for TMS delivered during baseline or rest is not included; For each publication, only key/novel findings are reported; ppTMS protocols require also administering single TMS pulses, yet only ppTMS-related characteristics/findings are inserted.

**Table 3:** Characteristics of key studies using TMS in motor decision making.

Reference	Task and Features	Task-Related TMS Timings	TMS Location	Main Findings and Elements of Novelty
Klein et al. (2012) [33]	Hand selection task with ambiguous and unambiguous trials	Go signal + 0.17, 0.33, 0.50, 0.67 × 66% of individual median RT	Right M1	Larger left MEPs in the reward_biased_, compared to reward_neutral_ (especially in ambiguous trials); link between reward-induced effects on MEP and movement preferences
Klein-Flügge & Bestmann (2012) [36]	Value-decision task with choice and forced choice trials	Forced choice trials: 10%, 35%, 50%, 60%, 70%, 80% of individual mean forced choice RT (FC-RT). Choice trials: 10%, 45% FC-RT, 45% FC-RT + 0.25*RT difference between choice and forced choice trials (ΔRT), 45% FC-RT + 0.5*ΔRT, 45% FC-RT + 0.75*ΔRT, 45% FC-RT + ΔRT	Left M1	MEPs differences between selected and non-selected muscles during the decision period in choice trials; MEPs in choice trials vary as a function of the expected value difference for alternative responses
Cos et al. (2014) [34]	Reach-decision task for movements with different biomechanical costs	1, 150, 200, 250, 300, 350 ms after stimuli onset	M1	The predicted cost associated with action alternatives is reflected in MEP changes (larger MEPs for less effortful movements early in the trial)
Mooshagian et al. (2015) [38]	Decision-making task manipulating reward probability/uncertainty and task framing	250 ms after stimuli onset	Left M1	MEPs linearly increase with reward probability in the find condition; varying the degree of outcome uncertainty does not result in MEPs modulation
Derosiere et al. (2022) [35]	Tokens task with rewards and penalties	After 1, 4, 7 token jumps	Right and left M1	Hasty motor decisions are supported by a broad motor facilitation in the selected body side together with a local suppression of motor representations surrounding the selected effector

RT = reaction time; M1 = primary motor cortex; MEP = motor evoked potential. Note: Studies are listed in chronological order; Only task-specific TMS timings are reported—information for TMS delivered during baseline or rest is not included; For each publication, only key/novel findings are reported.
